# Complete Genome Sequence of Lactobacillus crispatus AB70, Isolated from a Vaginal Swab from a Healthy Pregnant Korean Woman

**DOI:** 10.1128/MRA.01736-18

**Published:** 2019-03-21

**Authors:** Dong-Ho Chang, Moon-Soo Rhee, Sung-Ki Lee, In-Hwa Chung, Haeyoung Jeong, Byoung-Chan Kim

**Affiliations:** a Metabolic Regulation Research Center, Korea Research Institute of Bioscience and Biotechnology (KRIBB), Daejeon, Republic of Korea; b Department of Biological Science and Biotechnology, Hannam University, Daejeon, Republic of Korea; c Korean Collection for Type Cultures (KCTC), Korea Research Institute of Bioscience and Biotechnology, Jeongeup-si, Jeollabuk-do, Republic of Korea; d Department of Obstetrics and Gynecology, College of Medicine, Myunggok Medical Research Center, Konyang University, Daejeon, Republic of Korea; e Sungwun Pharmacopia Co., Ltd., Seongnam-si, Gyeonggi-do, Republic of Korea; f Infectious Disease Research Center, KRIBB, Daejeon, Republic of Korea; g Department of Biosystems and Bioengineering, KRIBB School of Biotechnology, Korea University of Science and Technology (UST), Daejeon, Republic of Korea; h Department of Bioprocess Engineering, KRIBB School of Biotechnology, UST, Daejeon, Republic of Korea; University of Maryland School of Medicine

## Abstract

The vaginal bacterial strain AB70, belonging to the species Lactobacillus crispatus, was isolated from a vaginal swab from a healthy pregnant Korean woman. Here, we report the 2.37-Mb complete genome sequence of this strain.

## ANNOUNCEMENT

The vaginal microbiota is known to play a significant role in women’s health and pregnancy (1, 2). Several studies have shown that the vaginal microbiome of healthy pregnant women is dominated by *Lactobacillus* spp. and is characterized by less richness and diversity than the microbiome of nonpregnant women (3). A poor dominance of vaginal lactobacilli may cause microbial imbalance in the vagina, often resulting in bacterial vaginosis and high-risk pregnancies (4).

To investigate vaginal microbiota using both culture-dependent and culture-independent techniques, vaginal swabs from healthy pregnant Korean women were collected under anaerobic conditions, as previously described (5). This study received ethical approval from the Konyang University Hospital Institutional Review Board (IRB) (approval number 2014-06-009). Using anaerobic de Man, Rogosa, and Sharpe (MRS) agar plates, several hundred purified vaginal microbes were isolated to purity using single-colony isolation, and most were assigned within Lactobacillus crispatus based on 16S rRNA sequences (100% 16S rRNA gene sequence similarities to that of L. crispatus strain ATCC 43058^T^). The 16S rRNA gene was amplified by colony PCR with the universal PCR primers 27F and 1492R (6), and purified PCR products were sequenced by the BIOFACT Co., Ltd. (Daejeon, Republic of Korea).

Antibacterial activities of ethyl acetate extracts were evaluated with disk diffusion assays against Escherichia coli KCTC 2441 and Bacillus cereus KCTC 3624. One of the isolates, named strain AB70, displayed strong antibacterial activities and was selected for further investigation, including complete genome analysis.

The genomic DNA of strain AB70 was extracted from 1-liter anaerobic MRS liquid cultures, as previously described (5). Genomic library construction and sequencing were carried out at the Chun Lab (Seoul, Republic of Korea) using the PacBio RS II platform (Pacific Biosciences, Menlo Park, CA, USA) with P6-C4 chemistry. We produced 150,292 sequence reads (ca. 1.1 Gbp) from one single-molecule real-time sequencing (SMRT) cell, and these were filtered with default parameters (read quality, ≥0.75; read length, ≥50 bp) with SMRT Analysis version 2.3.0. The resulting 111,972 filtered reads (960.4 Mb total; average length, 8,576 bp) were assembled into two contigs with Canu (7) version 1.1 (parameters: genomeSize = 2.4 m, pacbio-raw) and circularized with Circlator (8) version 1.5.1. Residual errors were corrected by running two consecutive rounds of the RS_Resequencing.1 protocol in SMRT Analysis. Genome annotation was carried out with the NCBI Prokaryotic Genome Annotation Pipeline (9).

Lactobacillus crispatus AB70 has a chromosome (2,351,263 bp, 37.3% G+C content) that encodes 2,333 protein-coding sequences, 71 tRNAs, and 5 rRNA gene clusters, while the circular plasmid pLcAB70 (16,662 bp, 35.8% G+C content) encodes only 19 protein-coding sequences. The average nucleotide identity between AB70 and the type strain ATCC 33820 calculated with the OrthoANIu algorithm (10) was 97.5%. Genome coverages, however, were only 50.7% (AB70) and 60.0% (ATCC 33820), in accordance with the considerable genetic diversity between strains (11). The genome most similar to AB70 among all 57 publicly available L. crispatus genomes in the RefSeq database, identified with dRep (12) version 2.0.5, was that of strain UMB0803 (GenBank number GCF_002861765, 99.7% average nucleotide identity [ANI]), one of the nine L. crispatus strains isolated from female urine collected with a transurethral catheter (BioProject number PRJNA316969, 200 strains).

The L. crispatus AB70 genome contains no virulence-related or resistance-related genes, as assessed with IslandViewer 4 (13), which identified 266,727-bp putative genomic islands (11.3% of the chromosome), distributed into 17 regions. We also identified two bacteriocin biosynthetic gene clusters with antiSMASH version 4.1.0 (14), one of which was similar to a gassericin T (15) biosynthetic gene cluster.

The genomic information from strain AB70 will improve our understanding of the genetic diversity of the species L. crispatus, a major member of the vaginal microbiome of healthy pregnant women.

### Data availability.

The complete genome sequences of L. crispatus AB70 have been deposited in DDBJ/ENA/GenBank under the accession numbers CP026503 (chromosome) and CP026504 (plasmid). The versions described in this paper are versions CP026503.1 and CP026504.1. Raw sequencing reads are available in NCBI BioProject under the accession number PRJNA431864.
